# Effects of epigallocatechin-3-gallate on thermogenesis and mitochondrial biogenesis in brown adipose tissues of diet-induced obese mice

**DOI:** 10.1080/16546628.2017.1325307

**Published:** 2017-05-26

**Authors:** Mak-Soon Lee, Yoonjin Shin, Sunyoon Jung, Yangha Kim

**Affiliations:** ^a^ Department of Nutritional Science and Food Management, Ewha Womans University, Seoul, Republic of Korea

**Keywords:** EGC, Gthermogenesis, mitochondrial biogenesis, brown adipose tissues, obesity

## Abstract

**Background**: Epigallocatechin-3-gallate (EGCG) is the major polyphenol in green tea and has been considered a natural agent that can help to reduce the risk of obesity.

**Objective**: The aim of this study was to investigate the effects of EGCG on thermogenesis and mitochondrial biogenesis in brown adipose tissue (BAT) of diet-induced obese mice.

**Methods**: Male C57BL/6J mice were provided a high-fat diet for 8 weeks to induce obesity, following which they were divided into two groups: one on a high-fat control diet and the other on a 0.2% EGCG (w/w)-supplemented high-fat diet for another 8 weeks.

**Results**: The EGCG-supplemented group showed decreased body weight gain, and plasma and liver lipids. EGCG-fed mice exhibited higher body temperature and mitochondrial DNA (mtDNA) content in BAT. The messenger RNA levels of genes related to thermogenesis and mitochondrial biogenesis in BAT were increased by EGCG. Moreover, adenosine monophosphate-activated protein kinase (AMPK) activity in BAT was stimulated by EGCG.

**Conclusions**: The results suggest that EGCG may have anti-obesity properties through BAT thermogenesis and mitochondria biogenesis, which are partially associated with the regulation of genes related to thermogenesis and mitochondria biogenesis, and the increase in mtDNA replication and AMPK activation in BAT of diet-induced obese mice.

## Introduction

Brown adipose tissue (BAT) has been identified as an important site for energy expenditure in the form of thermogenesis. Because it has high metabolic capacity to maintain body temperature in cold conditions or to waste food energy, it is currently considered a novel therapeutic option for obesity management. BAT thermogenesis is principally dependent on the activation of uncoupling proteins (UCPs), which causes conversion of the driving force of adenosine triphosphate (ATP) synthesis into heat via uncoupling oxidative phosphorylation in the mitochondrial inner membrane [1]. In response to cold temperatures, sympathetic nervous stimulation, and β-adrenergic agonist administration, the activities of UCP increase rapidly [2]. This heat-producing mechanism in BAT is referred to as ‘non-shivering’ thermogenesis, which is in contrast to ‘shivering’ thermogenesis in muscle tissues. It is assumed that a defect in BAT mitochondria causes faculty non-shivering thermogenesis in obesity [3].

At a cellular level, one of the important processes of BAT thermogenesis is the biogenesis of mitochondria. Puigserver et al. [4] observed that cold or adrenergic stimulation increased the expression of the peroxisome proliferator-activated receptor gamma coactivator-1α (PGC-1α) gene in BAT. PGC-1α is known as the primary stimulator of mitochondrial biogenesis. It provokes the activation of nuclear respiratory factor-1 (NRF1) and mitochondrial transcription factor A (Tfam) that enhance the expression of genes required for mitochondrial function, such as mitochondrial transcription factors specific for mitochondrial DNA (mtDNA) [5,6]. These molecules may act to induce high mitochondrial density in BAT and contribute to effective BAT thermogenesis.

Previous studies have indicated that green tea extract and catechin-polyphenols stimulate fat oxidation, energy expenditure, and thermogenesis in humans and rats [7,8]. A recent study reported that a green tea extract containing epigallocatechin-3-gallate (EGCG) and caffeine stimulated non-shivering thermogenesis and increased energy expenditure during cold exposure in humans [9]. Green tea polyphenolic extracts have also been shown to improve renal function by stimulating mitochondrial biogenesis in rat kidneys [10]. The stimulation of mitochondrial biogenesis was associated with the induction of PGC-1α and Tfam gene expression, and mtDNA replication. A few studies have investigated the effects of EGCG-containing green tea extracts on thermogenesis and mitochondrial biogenesis; however, the effects of EGCG on BAT in diet-induced obese mice remain unknown.

Our study focused on the effect of EGCG on thermogenesis and mitochondrial biogenesis in BAT of diet-induced obese mice. Thus, we measured the body temperature of mice during cold exposure. In addition, messenger RNA (mRNA) levels of genes related to thermogenesis and mitochondrial biogenesis, mtDNA copy number, and adenosine monophosphate-activated protein kinase (AMPK) activity in BAT of diet-induced obese mice were evaluated.

## Methods and materials

### Animals and treatments

Twelve male C57BL/6J mice, 4 weeks old, were obtained from Charles River (Hino, Japan) and individually housed in stainless steel wire mesh cages in a room maintained at 22 ± 1°C with a 12 h light/dark cycle (light period: 08:00–20:00 h). They were fed laboratory chow and had access to water *ad libitum* for 1 week to stabilize their metabolic condition. To induce obesity in the mice, they were fed a high-fat diet (45% of total energy), containing 23% (w/w) fat, 17% (w/w) casein, 12% (w/w) sucrose, 20% (w/w) starch, 15% (w/w) dextrose, 4% (w/w) cellulose, 4.3% (w/w) minerals, and 1.2% (w/w) vitamins. The diets were based on a modification of the AIN-93 diet and supplied gratis by Dyets (Bethlehem, PA, USA). After 8 weeks, the mice were randomly divided into two groups with six animals each and subsequently maintained on one of the following diets: a high-fat control diet or a high-fat diet supplemented with 0.2% (w/w) EGCG (TEAVIGO™; DSM Nutritional Products, Basel, Switzerland). The mice were maintained on these diets for an additional 8 weeks. Body weight and food intake were monitored twice a week. At the end of the experiment, the mice were starved overnight and anesthetized with ketamine/xylazine. A central longitudinal incision was made in the abdominal wall and blood samples were collected by cardiac puncture. Blood samples were centrifuged at 1500 × *g* for 20 min at 4°C and the plasma was separated and stored at −20°C until analyzed. The liver, white adipose tissue (WAT) and BAT were harvested, frozen immediately in liquid nitrogen, and stored at −70°C. All animal procedures conformed to the National Institutes of Health (NIH) guidelines as stated in the Principles of Laboratory Animal Care [11].

### Plasma biochemical measurements

The plasma levels of triacylglycerol (TG), total cholesterol (TC), aspartate transaminase (AST), and alanine transaminase (ALT) were determined by enzymic colorimetric methods using commercial kits (Asan Pharmaceutical, Seoul, Republic of Korea) in accordance with the manufacturer’s instructions. In these assays, reacted substrates produce colored end products and the color intensity of the end products was considered to be directly proportional to concentrations of TG, TC, AST, or ALT. Plasma leptin level was measured using a mouse/rat leptin enzyme-linked immunosorbent assay (ELISA) kit (R&D Systems, Minneapolis, MN, USA), which used the quantitative sandwich enzyme immunoassay technique.

## Liver and fecal lipid analysis

Liver and fecal lipids were extracted using the method of Bligh and Dyer [12], with slight modifications [13]. TG and TC concentrations were determined by enzymic colorimetric methods using commercial kits (Asan Pharmaceutical, Seoul, Republic of Korea) in accordance with the manufacturer’s instructions.

### Measurement of body temperature

Body temperature was measured in mice as described previously [13]. Mice were fasted for 16 h after 8 weeks on a control diet or an EGCG-supplemented diet. They were exposed to a temperature of 4°C for up to 6 h. Core body temperature was measured during the cold exposure period with a digital thermometer (Testo 925).

### mtDNA content analysis

The amount of mtDNA was determined according to previous work [14]. The genomic DNA was detected in BAT by using a Puregene DNA isolation kit (Qiagen, Valencia, CA, USA). mtDNA content was assessed by real-time quantitative polymerase chain reaction (qPCR) as the ratio of mitochondrial gene [cytochrome oxidase subunit I (Cox1)] to nuclear gene [glyceraldehyde-3-phosphate dehydrogenase (GAPDH)].

### Real-time qPCR

Total RNA was extracted from BAT using TRIzol Reagent (Invitrogen, Carlsbad, CA, USA). The corresponding complementary DNA (cDNA) was synthesized from 4 μg RNA using Moloney murine leukemia virus (M-MLV) reverse transcriptase (Invitrogen). After cDNA synthesis, real-time qPCR was performed using Universal SYBR Green PCR Master Mix (Qiagen, Chatsworth, CA, USA) in a fluorometric thermal cycler (Corbett Research, Mortlake, Australia). Primers were designed by the program Primer3 [15]. Sequences of the sense and antisense primers used for amplification are shown in Table 1. The ^∆∆^Ct method was used for relative quantification [16]. The ^∆∆^Ct value for each sample was determined by calculating the difference between the Ct value of the target gene and the Ct value of β-actin as a reference gene. The normalized level of expression of the target gene in each sample was calculated using the formula 2 − ^∆∆^Ct. Values were expressed as fold change relative to the control group.

**Table 1.** Primers used for the real-time quantitative polymerase chain reaction.

Name	GeneBank no.	Primer sequence (5ʹ–3ʹ)
ACC2	NM_133904	F: CACGAGATTGCTTTCCTAGG
		R: CAGGGTAAGGTTGGGATTTG
β-Actin	NM_007393	F: GGACCTGACAGACTACCTCA
		R: GTTGCCAATAGTGATGACCT
CPT-1β	NM_009948	F: CTCCGAAAAGCACCAAAACA
		R: CTCCAGCACCCAGATGATTG
NRF1	NM_010938	F: AAGTATTCCACAGGTCGGGG
		R: TGGTGGCCTGAGTTTGTGTT
PGC-1α	NM_008904	F: GGGCCAAACAGAGAGAGAGG
		R: GTTTCGTTCGACCTGCGTAA
PRDM16	BC_059838	F: GTCGGATGGCAGTGACTTTG
		R: AGGTTTGCTCTCCACTGGCT
Tfam	NM_009360	F: GAGGCCAGTGTGAACCAGTG
		R: GTAGTGCCTGCTGCTCCTGA
UCP1	NM_009463	F: CAGGCTTCCAGTACCATTAG
		R: CTTGGACTGAGTCGTAGAGG
UCP2	NM_011671	F: CAGAGCAGGAGGTTACAGTC
		R: TCAACCCCTTCATTACAGAC

ACC2, acetyl-coenzyme A carboxylase-2; CPT-1β, carnitine-palmitoyl-coenzyme A transferase-1β; NRF1, nuclear respiratory factor-1; PGC-1α, peroxisome proliferator-activated receptor gamma coactivator-1α; PRDM16, PR domain containing 16; Tfam, mitochondrial transcription factor A; UCP1, uncoupling protein 1; UCP2, uncoupling protein 2.

### AMPK activity assay

AMPK activity was evaluated using an AMPK Kinase Assay kit (Cyclex, Nagano, Japan), as previously described [13]. In brief, samples were incubated for 30 min at 30°C on a plate precoated with a substrate peptide corresponding to mouse insulin receptor substrate-1 (IRS-1). AMPK activity was detected by measuring the phosphorylation of Ser789 on IRS-1 with anti-mouse phospho-Ser789 IRS-1 monoclonal antibody and peroxidase-coupled anti-mouse immunoglobulin G, which catalyzes conversion of the chromogenic substrate tetramethylbenzidine. Protein was determined using a bicinchoninic acid (BCA) protein assay kit (Thermo Scientific; Waltham, MA, USA). AMPK activity was normalized to protein concentration and expressed as fold change relative to the control group.

### Statistical analysis

All data are presented as mean ± standard error of the mean (SEM). Significant differences between the two groups were analyzed by Student’s *t* test using SPSS software (version 17; IBM Corporation, Armonk, NY, USA). *p* < 0.05 was taken to indicate a statistically significant difference.

## Results

### Body weight, energy intake, and fat accumulation

Initial body weight did not differ between the two groups at the outset of the experiment. After 8 weeks of treatment, final body weight decreased by 11% in the EGCG-supplemented group compared with that in the control group (Table 2). Food and energy intakes were not different between the two groups; however, food and energy efficiency were significantly lower in the EGCG-supplemented group than in the control group. The weights of WAT and BAT were decreased by 45% and 34%, respectively, in the EGCG-supplemented group compared with those of the control group.

**Table 2.** Physiological variables of mice fed control or epigallocatechin-3-gallate (EGCG) diets for 8 weeks.

Variable	Control	EGCG
Initial body weight (g)	29.04 ± 0.71	29.04 ± 1.36
Final body weight (g)	43.76 ± 0.59	38.97 ± 0.84**
Body weight gain (g/8 weeks)	14.72 ± 0.57	9.93 ± 0.95**
Food intake (g/day)	3.27 ± 0.05	3.12 ± 0.07
Food efficiency (g gain/g consumed)	0.087 ± 0.003	0.061 ± 0.006**
Energy intake (kcal/day)	15.16 ± 0.21	14.48 ± 0.34
Energy efficiency (g gain/kcal consumed)	0.019 ± 0.001	0.013 ± 0.001**
Liver weight (g/100 g body weight)	3.36 ± 0.08	3.35 ± 0.09
Epididymal adipose tissue weight (g/100 g body weight)		
White adipose tissue	5.73 ± 0.16	3.14 ± 0.36***
Brown adipose tissue	1.14 ± 0.05	0.76 ± 0.12*

Values are expressed as mean ± SEM (*n* = 6).

Control, high-fat diet; EGCG, 0.2% EGCG-supplemented high-fat diet.

*p < 0.05, **p < 0.01, ***p < 0.001 vs control group.

### Plasma, liver, and fecal metabolites

The EGCG-supplemented group showed significantly decreased plasma TG (35%) and TC (25%) compared with the control group (Table 3). Liver lipid, TG, and TC levels significantly decreased, by 31%, 30%, and 31%, respectively, in the EGCG-supplemented group compared with those in the control group. The amounts of fecal lipid, TG, and TC in the EGCG-supplemented group increased by 1.4-fold, 2.2-fold, and 2.5-fold, respectively, compared with those in the control group. Plasma leptin level decreased by 65% in the EGCG-supplemented group compared with that in the control group.

**Table 3.** Plasma, liver, and fecal metabolites of mice fed control or epigallocatechin-3-gallate (EGCG) diets for 8 weeks.

Metabolite	Control	EGCG
Plasma lipids		
TG (mmol/l)	1.01 ± 0.05	0.65 ± 0.05***
TC (mmol/l)	4.60 ± 0.16	3.44 ± 0.18**
Plasma leptin (ng/ml)	16.53 ± 0.68	10.82 ± 0.60***
Plasma AST (IU/l)	70.50 ± 3.71	68.99 ± 4.93
Plasma ALT (IU/l)	19.02 ± 1.83	18.58 ± 2.58
Liver lipids		
Total lipid (μmol/g)	104.70 ± 4.91	71.36 ± 4.81**
TG (μmol/g)	19.26 ± 2.18	13.56 ± 0.73*
TC (μmol/g)	20.03 ± 1.50	13.75 ± 0.78**
Fecal lipids		
Total lipid (μmol/g)	42.36 ± 2.46	61.00 ± 4.90**
TG (μmol/g)	0.82 ± 0.16	1.84 ± 0.26**
TC (μmol/g)	2.66 ± 0.14	6.68 ± 0.51***

Values are expressed as mean ± SEM (*n* = 6).

Control, high-fat diet; EGCG, 0.2% EGCG-supplemented high-fat diet; TG, triglyceride; TC, total cholesterol; AST, aspartate aminotransferase; ALT, alanine aminotransferase.

*p < 0.05, **p < 0.01, ***p < 0.001 vs control group.

### Liver weight and plasma AST and ALT activities

To investigate whether EGCG induced hepatic toxicity, we measured liver weight and plasma AST and ALT activities. The activities of plasma AST and ALT were within the normal range in mice, at 68.99–70.50 IU/l and 18.58–19.02 IU/l, respectively (Table 3). There were no significant differences between the groups regarding liver weight (Table 2) and plasma AST and ALT levels (Table 3).

### Body temperature

**Figure 1.**
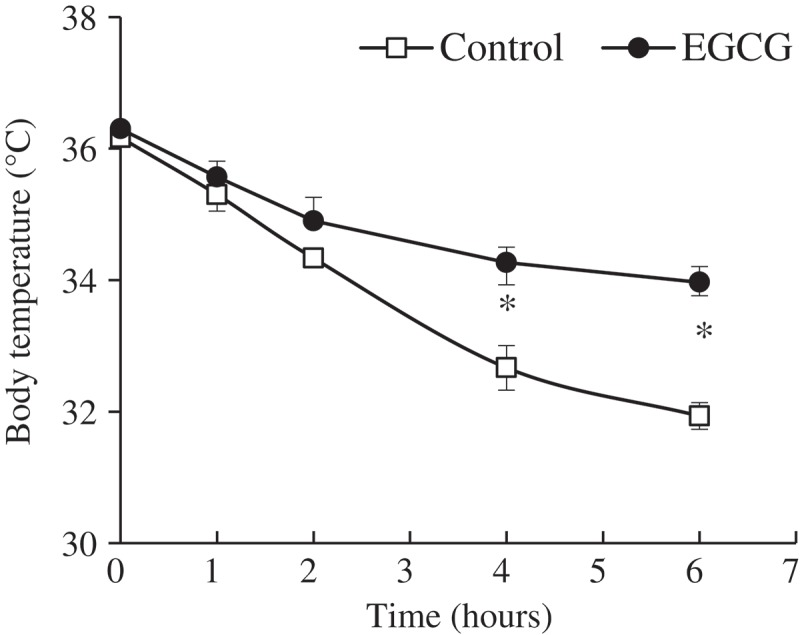
Body temperature of mice fed control or epigallocatechin-3-gallate (EGCG) diets for 8 weeks. After 8 weeks of dietary supplementation, body temperature was measured during exposure to 4°C for 6 h. Values are expressed as mean ± SEM (*n* = 6). *p < 0.05 vs control group. Control, high-fat diet; EGCG, 0.2% EGCG-supplemented high-fat diet.

A cold test was performed after 8 weeks of EGCG supplementation to estimate the effects on thermogenesis. Core body temperature was significantly higher in the EGCG-supplemented group than in the control group at 4 h and 6 h of cold exposure (Figure 1).

### mtDNA content in BAT

**Figure 2.**
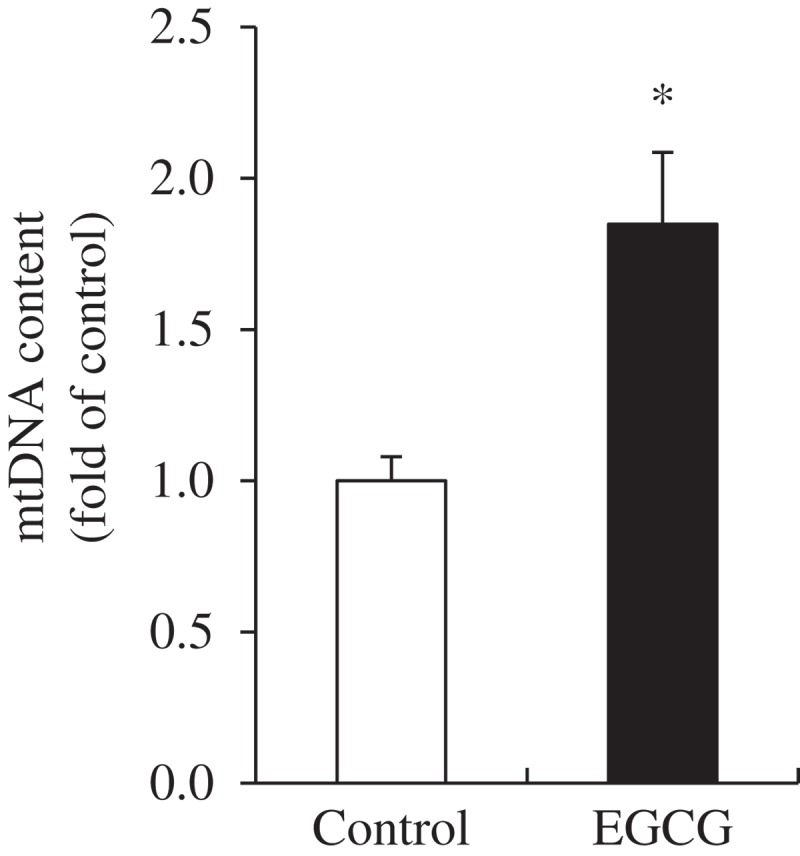
Mitochondrial DNA (mtDNA) content in brown adipose tissue of mice fed control or epigallocatechin-3-gallate (EGCG) diets for 8 weeks. Values for the EGCG group are expressed as the fold change compared with those for the control group (mean ± SEM, *n* = 6). *p < 0.05 vs control group. Control, high-fat diet; EGCG, 0.2% EGCG-supplemented high-fat diet.

To evaluate the effect of EGCG on mitochondrial biogenesis in BAT, mtDNA content was measured. The mtDNA content significantly increased in EGCG-supplemented group (1.85-fold) compared with that of the control group (Figure 2).

### mRNA levels of genes involved in thermogenesis and mitochondrial biogenesis in BAT

**Figure 3.**
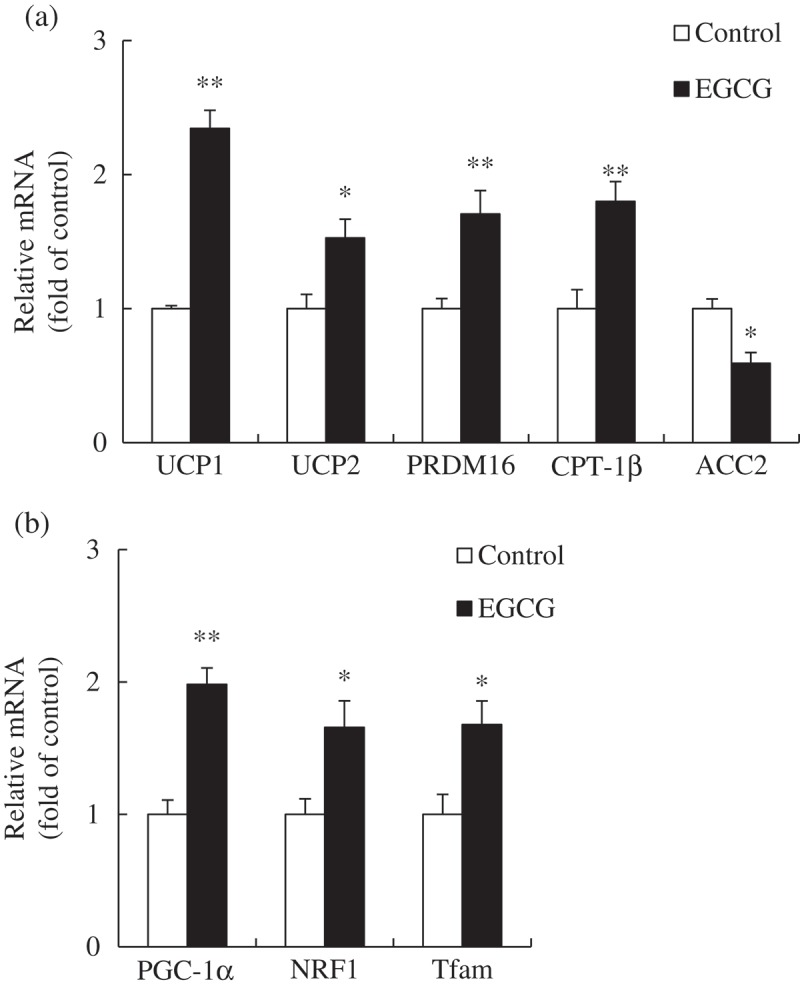
Relative messenger RNA (mRNA) levels of genes involved in (a) thermogenesis and (b) mitochondrial biogenesis in brown adipose tissue of mice fed control or epigallocatechin-3-gallate (EGCG) diets for 8 weeks. Values for the EGCG group are expressed as the fold change compared with those for the control group (mean ± SEM, *n* = 6). *p < 0.05, **p < 0.01 vs control group. Control, high-fat diet; EGCG, 0.2% EGCG-supplemented high-fat diet. UCP1, uncoupling protein 1; UCP2, uncoupling protein 2; PRDM16, PR domain containing 16; CPT-1β, carnitine-palmitoyl-coenzyme A transferase-1β; ACC2, acetyl-coenzyme A carboxylase-2; PGC-1α, peroxisome proliferator-activated receptor gamma coactivator-1α; NRF1, nuclear respiratory factor-1; Tfam, mitochondrial transcription factor A.

To determine the relationship between EGCG supplementation and the increase in body temperature, the mRNA levels of genes related to thermogenesis such as UCP1, UCP2, PR domain containing 16 (PRDM16), carnitine-palmitoyl-coenzyme A transferase-1β (CPT-1β), and acetyl-coenzyme A carboxylase-2 (ACC2) were measured in BAT. Levels of mRNA for PGC-1α, NRF1, and Tfam were also measured to investigate the effects of EGCG on mitochondrial biogenesis in BAT. Dietary EGCG significantly increased the mRNA levels of UCP1, UCP2, PRDM16, and CPT-1β, while decreasing the mRNA level of ACC2, compared with those in the control group (Figure 3(a)). The mRNA levels of PGC-1α, NRF1, and Tfam were noticeably increased in the EGCG-supplemented group compared with those in the control group (Figure 3(b)).

### AMPK activity in BAT

**Figure 4.**
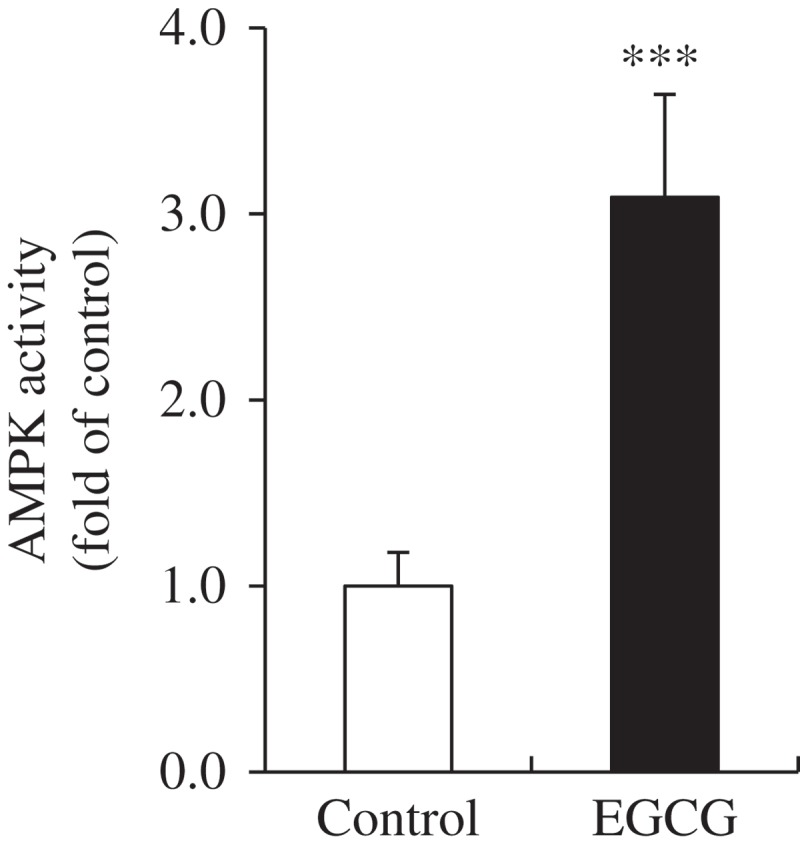
AMP-activated protein kinase (AMPK) activity in brown adipose tissue of mice fed control or epigallocatechin-3-gallate (EGCG) diets for 8 weeks. Values for the EGCG group are expressed as the fold change compared with those for the control group (mean ± SEM, *n* = 6). ***p < 0.001 vs control group. Control, high-fat diet; EGCG, 0.2% EGCG-supplemented high-fat diet.

We determined the AMPK activity in BAT, which affects the mRNA expression of genes related to thermogenesis and mitochondrial biogenesis. The EGCG-supplemented group showed a 3.1-fold increase in AMPK activity compared with the control group (Figure 4).

## Discussion

In this study, we investigated the anti-obesity effects of dietary EGCG based on molecular factors involved in thermogenesis and mitochondrial biogenesis in the BAT of high-fat diet-induced obese mice. The EGCG dosage (0.2% w/w) used in our study was in the non-hepatotoxic range for mice, as demonstrated by the unaffected plasma levels of AST and ALT during EGCG supplementation. Following the EGCG diet, body weight, as well as WAT and BAT mass, noticeably reduced. Plasma and liver lipids also decreased, while fecal lipid excretion increased. Energy intake did not differ between the two groups. The anti-obesity actions of EGCG have been explained by lipid modulation in WAT. WAT is the major site of energy storage and hormone release that controls whole-body metabolism. In our previous study [17], we reported reduction in body weight and adipose tissue mass after green tea-EGCG supplementation in diet-induced obese mice. These effects were related to the suppression of adipogenic genes and the stimulation of genes involved in lipolysis, β-oxidation, and thermogenesis in WAT. Wolfram et al. [18] also observed that EGCG supplementation decreased subcutaneous and epididymal adipose tissue weights as well as mRNA expression of genes involved in fatty acid synthesis in epididymal adipose tissue of C57BL/6J mice.

BAT is an essential organ for thermogenesis, which is the increase in energy expenditure in response to diet. During thermogenesis, BAT uses fatty acids to generate heat, which results from the action of UCPs. To understand the mechanisms underlying BAT thermogenesis with EGCG supplementation, we measured body temperature during a mild cold challenge and the mRNA levels of the genes related to thermogenesis, such as UCP1, UCP2, PRDM16, CPT-1β, and ACC2 in BAT. UCPs are mitochondrial inner membrane proteins that uncouple oxidative respiration, and three such proteins have been reported thus far [19]. UCP1 is mainly expressed in BAT [20,21], UCP2 is ubiquitously expressed in various tissues in the body [22,23], and UCP3 is specific for skeletal muscle and BAT [24]. PRDM16 is a dominant regulator of brown fat development, and reduction in its expression has been associated with a decrease in the expression of UCP1 [25]. CPT-1β is a rate-limiting enzyme in the regulation of fatty acid uptake and oxidation by mitochondria in BAT. Mice lacking CPT-1β died in response to a cold challenge owing to their inability to generate heat [26], which implies that CPT-1β plays an important role in stimulating BAT thermogenesis. On the other hand, ACC2 inhibits activity of CPT-1β by catalyzing the formation malonyl-coenzyme (CoA) from acetyl-CoA [27]. Dulloo et al. [7] demonstrated increases in 24 h energy expenditure by supplementation of green tea extract in humans. Subsequently, they reported an enhancement in the oxygen uptake rate for BAT by administering an ethanol extract of green tea, indicating the stimulation of BAT thermogenesis [8]. The body fat-suppressive effect of green tea extract increased BAT thermogenesis through β-adrenoreceptor activation in rats fed a high-fat diet [28]. Green tea EGCG attenuates diet-induced obesity by stimulating UCP2 expression in WAT [17]. On the other hand, it has been reported that dietary garlic increases the body temperature and UCP1 expression in BAT of mice fed a high-fat diet [13]. Zhang et al. [29] reported that berberine activates thermogenesis in WAT and BAT. Berberine has been shown to enhance mRNA expression of UCP1 and CPT1, while increasing protein expression of UCP1 and CPT1 in db/db mice. Orally administered *Aster spathulifolius* extract increased the mRNA and protein expression of genes involved in thermogenesis such as CPT1, UCP2, and UCP3, while decreasing ACC2 expression in the muscle of HFD-induced obese rats [30]. We found that expression of UCP1, UCP2, PRDM16, and CPT-1β in BAT up-regulated significantly upon EGCG supplementation, whereas the ACC2 expression was down-regulated, which was consistent with the observed increase in body temperature during cold acclimation. These results suggest that dietary EGCG may enhance thermogenesis through regulation of the expression of BAT thermogenic genes with increased body temperature in diet-induced obese mice.

Mitochondria regulate whole-body energy metabolism by controlling energy expenditure and heat generation in BAT. Mitochondrial biogenesis is crucial for cellular health, and is regulated by PGC-1α, NRF1, and Tfam genes [31,32]. PGC-1α is a co-transcriptional regulation factor that induces mitochondrial biogenesis by activating NRF1 [32]. NRF1 has been linked to the expression of genes involved in mitochondrial function and biogenesis. The NRF1 gene induces Tfam expression, which regulates mtDNA copy number and transcriptional activity [31]. In this study, dietary EGCG increased the mtDNA copy number and expression of PGC-1α, NRF1, and Tfam genes in the BAT of diet-induced obese mice. We believe that higher mRNA expression of PGC-1α, NRF1, and Tfam was relevant to a higher production of those proteins and contributed to the increased mtDNA content. In a previous study, acute exercise stimulated PGC-1α and Tfam protein expression while increasing mRNA expression of genes including PGC-1α, Tfam, NRF1, and COX-1 [33]. Moreover, mice fed a high-fat diet showed a reduction in muscle mtDNA content as well as reduced muscle PGC-1α mRNA and protein expression [34]. Berberine induced thermogenesis in db/db/mice by stimulating BAT activity, as evidenced by increased mtDNA copy number as well as increased protein and mRNA expression of genes including PGC-1α and NRF1 in BAT [29]. In line with those studies, Rehman et al. [10] reported that green tea polyphenols improve renal function by enhancing mitochondrial biogenesis. The green tea polyphenols have been shown to increase mtDNA copy number as well as mRNA and protein expression of PGC-1α and Tfam in rat kidneys. Thus, it can be postulated that EGCG may induce mitochondrial biogenesis by stimulating mtDNA replication and expression of genes involved in mitochondrial biogenesis in BAT of diet-induced obese mice.

AMPK activation can regulate energy metabolism that favors the stimulation of catabolic pathways by enhancing oxidative metabolism and mitochondrial biogenesis [35]. These metabolic processes inactivate key enzymes in lipid biosynthetic pathways, such as acetyl-CoA carboxylase [36]. AMPK has been shown to control PGC-1α and mitochondrial enzyme gene expression in skeletal muscle [37] and epididymal adipose tissue [38]. Mulligan et al. [39] showed that AMPK activity increased in BAT of the mouse under chronic cold exposure, suggesting its role in regulating thermogenesis. Inokuma et al. [40] also showed that AMPK activation was stimulated by norepinephrine (noradrenaline) in BAT of wild-type mice, and not in UCP1-knockout mice. Several data suggested that AMPK activity is associated with increased PGC-1α-mediated mitochondrial biogenesis in skeletal muscle [41,42]. This phenomenon raises the possibility that AMPK activation contributes to the high mitochondrial density in BAT. AMPK was shown to control cyclooxygenase-2 in EGCG-treated colon cancer cells [43]. In particular, EGCG has been reported to activate AMPK and to reduce endothelin-1 (ET-1) expression in vascular endothelial cells. The reduction of ET-1 expression was inhibited by the AMPK inhibitor Compound C [44]. In our study, we found that EGCG supplementation increased AMPK activity in the BAT of diet-induced obese mice. Thus, it is assumed that EGCG was partially associated with AMPK activation on the thermogenesis and mitochondrial biogenesis of BAT.

## Conclusion

**Figure 5.**
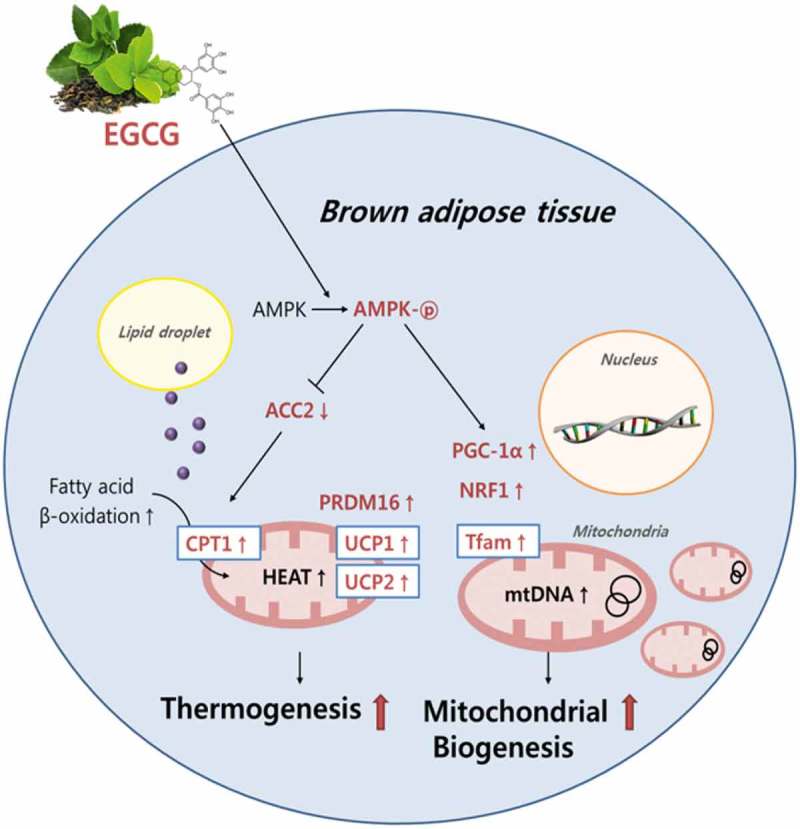
Schematic diagram showing the possible mechanisms(s) of brown adipose tissue thermogenesis and mitochondrial biogenesis by dietary epigallocatechin-3-gallate (EGCG) in diet-induced obese mice. AMPK, AMP-activated protein kinase; ACC2, acetyl-coenzyme A carboxylase-2; carnitine-palmitoyl-coenzyme A transferase-1; UCP1, uncoupling protein 1; UCP2, uncoupling protein 2; PRDM16, PR domain containing 16; PGC-1α, peroxisome proliferator-activated receptor gamma coactivator-1α; NRF1, nuclear respiratory factor-1; Tfam, mitochondrial transcription factor A; mtDNA, mitochondrial DNA.

It is apparent that dietary EGCG decreased body weight and plasma and liver lipid profiles, and increased fecal lipid excretion in the diet-induced obese mice. These actions of EGCG were mediated, at least partially, by the regulation of genes related to thermogenesis and mitochondrial biogenesis of BAT with an enhancement of body temperature. It is also likely that EGCG is directly linked to the increase in mtDNA replication and AMPK activation in BAT (Figure 5). We assume from these results that EGCG may have the ability to prevent obesity, which is partially associated with the regulation of the expression of multiple genes during thermogenesis and mitochondrial biogenesis, and the induction of mtDNA replication and AMPK activation in BAT of diet-induced obese mice. These findings suggest that EGCG may play important roles in regulating BAT thermogenesis and mitochondrial biogenesis for improving obesity.
